# Development of a Moderated Online Intervention to Treat Social Anxiety in First-Episode Psychosis

**DOI:** 10.3389/fpsyt.2019.00581

**Published:** 2019-08-14

**Authors:** Carla McEnery, Michelle H. Lim, Ann Knowles, Simon Rice, John Gleeson, Simmone Howell, Penni Russon, Chris Miles, Simon D’Alfonso, Mario Alvarez-Jimenez

**Affiliations:** ^1^Centre for Mental Health, Swinburne University of Technology, Hawthorn, VIC, Australia; ^2^Centre for Youth Mental Health, The University of Melbourne, Parkville, VIC, Australia; ^3^Orygen, The National Centre of Excellence in Youth Mental Health, Parkville, VIC, Australia; ^4^Iverson Health Innovation Research Institute, Swinburne University of Technology, Hawthorn, VIC, Australia; ^5^Department of Psychological Sciences, Swinburne University of Technology, Hawthorn, VIC, Australia; ^6^School of Behavioural and Health Sciences, Australian Catholic University, Melbourne, VIC, Australia; ^7^School of Computing and Information Systems, University of Melbourne, Parkville, VIC, Australia

**Keywords:** social anxiety, social phobia, psychosis, online psychosocial interventions, schizophrenia spectrum and other psychotic disorder

## Abstract

**Background:** It is well established that social anxiety disorder (SAD) is a significant clinical problem for individuals with a psychotic disorder. Comorbid social anxiety in individuals with psychosis has been associated with poorer premorbid functioning, increased depression, and a reduced quality of life. Cognitive behavior therapy (CBT) is recommended for people with psychosis as a first-line psychological treatment; however, its focus and evaluation primarily revolves around reducing psychotic symptoms and not necessarily targeting comorbid social anxiety symptoms. We developed a novel online social cognitive behavioral intervention (entitled EMBRACE) specifically designed to treat social anxiety symptoms in first episode psychosis (FEP).

**Methods:** The key clinical and engagement features of the intervention were established through integrating evidence-based material derived from 1) CBT-based treatment models for SAD, 2) relevant literature findings related to psychosis and its clinical correlates (e.g., shame, social rank, and its relationship with social anxiety and paranoia), 3) feedback from youth focus groups in order to inform a user-centered intervention design, and 4) a highly multidisciplinary collaborative development approach to design therapy comics.

**Results:** A detailed description of the final version of the 12-week online social intervention to treat social anxiety in FEP is presented.

**Conclusion:** The EMBRACE intervention was designed to provide young people with the necessary skills and confidence to overcome social anxiety within a supportive, safe online space. By design, it allows young people the opportunity to practice their newly learnt skills to connect with others and in doing so, learn to *embrace* their true authentic selves.

## Introduction

Social anxiety disorder (SAD) is recognized as one of the most common anxiety disorders, with a reported lifetime prevalence of 13.3% and a 12-month prevalence rate of 2.3% (1–5). Empirical findings indicate that SAD is the third most common psychiatric disorder after depression (17%) and alcohol dependence (14%) (1–5). It is now well established that SAD is also a significant problem for people with psychotic disorder diagnosis (6–10). A 2019 meta-analysis reported SAD prevalence rates of 25% for individuals with psychotic disorder diagnosis (6), and a growing body of evidence suggests that SAD is a relatively common occurrence following a first episode of psychosis (FEP) (10, 11). Study findings have reported that, in the first year following the onset of psychosis, up to 29% of young adults experience symptoms that meet diagnostic criteria for SAD (10, 11).

There is evidence to suggest that SAD is not simply a phenocopy arising from psychotic symptoms, such as paranoia and/or social anhedonia, and in fact, constitutes a distinct comorbid clinical presentation requiring a specific clinical focus (8, 10, 11). For instance, empirical findings indicate several observed differences between individuals with a psychotic disorder diagnosis compared to those individuals with comorbid psychotic disorder and SAD diagnostic presentations (8). These observed clinical differences include greater levels of subjective shame (11, 12), greater tendencies towards personalization bias or self-blame (12–16), greater perceptions of lower social rank (13, 15, 17), and lower levels of self-esteem (13, 14) in individuals with comorbid psychotic disorder and SAD diagnoses compared to those with psychotic disorder diagnoses only.

Comorbid SAD is clinically important, as lower overall functioning and a higher risk for suicide attempts have also been observed in individuals with comorbid psychotic disorder and SAD diagnoses when compared to individuals with psychotic disorder diagnoses only (8, 12–16). Findings also show that individuals with a FEP diagnosis and SAD comorbidity exhibit poorer early adjustment and are more prone to relapse (10–12) in comparison to individuals with a FEP diagnosis only. Taken together, these findings indicate that the treatment of comorbid SAD in individuals with a psychotic disorder diagnosis warrants distinct research and clinical focus in order to address the myriad of negative functional consequences and subjective distress associated with SAD comorbidity among psychosis populations.

Cognitive behavior therapy for psychosis (CBT*p*) is recommended as a first-line psychological treatment for people with psychosis (National Institute for Health and Care Excellence: NICE (18, 19); however, its focus and evaluation primarily revolves around reducing psychotic symptoms and not treating affective comorbidity, such as social anxiety. Although CBT has been shown to be effective for the treatment of SAD in non-psychotic populations, there are limited studies that have evaluated the clinical effectiveness of CBT for the primary treatment of social anxiety when this is co-morbid among individuals with psychosis (20–22).

To the best of our knowledge, there have only been two randomized controlled trials (RCT) (23, 24) conducted to date that have specifically targeted SAD using CBT in psychosis populations. The findings from these trials indicate that such approaches are effective in reducing social anxiety symptomatology in individuals with comorbid psychosis and SAD (25). To date, and to the authors’ knowledge, there have been no CBT-based intervention studies that have specifically targeted SAD as a primary treatment concern in a FEP population. This is problematic, as early interventions offer an opportunity to reduce the incidence and thus the burden of social anxiety on individuals with a FEP (26). More research is required to assess the feasibility, acceptability, and clinical benefits of interventions utilizing tailored CBT techniques to treat social anxiety symptomatology in individuals along the psychosis continuum, in particular early interventions for ultra-high risk and FEP populations.

Online interventions hold great promise for transforming the delivery of tailored evidence-based treatment for social anxiety in psychosis (27). This technology is particularly pertinent in the delivery of FEP interventions, as individuals under 25 years are the highest users of internet-based resources, with 92% reporting online daily usage (27, 28). Research findings also show that up to a quarter of this age group’s media usage (equating to over 6 h per week) is mainly spent on social networking sites (SNS) (27, 28). SNS are defined as online platforms that enable individuals to connect with other users to generate and maintain social connections (28, 29). Online interactions are particularly attractive to individuals who report problematic levels of social anxiety. Online interactions allow the individual a sense of increased control over self-presentation and, in doing so, can assist with averting the physical and cognitive symptoms of anxiety (29–32). Interactions *via* social networking, it could be argued, allow otherwise socially avoidant individuals a place where they can engage socially and foster connections, in addition to providing an opportunity to rehearse new skills and strategies in a safe way (33). In this way, a transitional social network can be developed that young people can use in their path to recovery (33).

Commercial SNS have also been the focus of controversy regarding their potential negative impact (34–37). Research findings indicate that young people who spend more than 2 h a day on SNS are more likely to report psychological distress than those spending less than 2 h a day, and that the more online social networks a young adult uses the more likely they are to report depressed mood and anxiety (35–37). The risks especially pertinent to a socially anxious population include the potential for unresolved miscommunication or committing a social faux pas, which can lead to repeated embarrassment, cyber-bullying, and reinforce maladaptive behaviors (38, 39). This can result in the maintenance of social anxiety symptoms together with increased social isolation and emotional vulnerability (37).

In order to harness the benefits of SNS while mitigating the purported risks, it is important that available online SNS for young socially anxious individuals provide opportunities for fostering meaningful engagements within a safe and non-stigmatizing environment. This requires engineering new kinds of online social networks to achieve this. One such way in which this could be achieved is *via* enabling social networking opportunities within an evidence-based online intervention, moderated by both peers and clinicians so that socially anxious individuals can interact safely within a therapeutic environment. In doing so, young people can be supported in applying their newly learned adaptive social behaviors to real-life, face-to-face social scenarios, thus harnessing the benefits of online social networking.

A particular consideration in developing a therapeutic online intervention that has social networking elements involves assisting socially anxious individuals, as needed, to overcome the tendency to passively consume content online rather than directly interact with other users (38). This tendency is a particular consideration for individuals with social anxiety, as evidence suggests that socially anxious individuals may use SNS passively (often referred to as “lurking”), rather than engaging interactively; research findings also show that online passive engagement may contribute to increased anxiety during future in-person interactions (38). Indeed, some researchers have argued that a combination of passive SNS usage, the increased use of safety behaviors (e.g., overly preparing online conversations), and the avoidance of in-person social interactions may perpetuate and increase social anxiety symptoms (37, 38).

In addition to this, although online interventions to treat social anxiety can be effective (36), poor adherence to online interventions is a common occurrence, and this is problematic given that high intervention adherence (relative to low intervention adherence) has been found to improve treatment outcomes (39). Therefore, in developing online evidence-based interventions to treat social anxiety in young people with FEP, a focus on 1) promoting direct interaction and 2) maximizing adherence to treatment (reducing attrition rate) is essential. Including persuasive technology elements, that is, intentionally designing online interventions to help change a participant’s voluntary attitudes or behavior (40), is one method by which this can be achieved. Persuasive technology is based on the theory of planned behavior (41) and the elaboration likelihood model (42). Both theories describe a path that involves changing a person’s behavior and attitude by influencing his or her motivation and beliefs (40, 43).

Persuasive technology techniques can be embedded within the design of online interventions to effectively and seamlessly support the goal of treating social anxiety symptomatology and to maximize users’ engagement potential (40). For example, a number of intervention features can be implemented to help achieve both of these goals such as providing: 1) tailored choices relating to assigned therapeutic tasks (i.e., tiered hierarchal options for the user to choose autonomously *via* the system), 2) dialogue support (i.e., automatic reminders, notifications from peers and clinicians), 3) credibility support (i.e., tailoring content relevant to the user *via* suggested material from moderators), and 4) social support (i.e., prompts to engage in social networking) (44).

The aim of this paper is to describe the development of an innovative, moderated online, social intervention, termed *EMBRACE*, designed to treat social anxiety as a primary treatment focus in young people with FEP. The web platform of the intervention was designed utilizing persuasive technology elements to promote participant engagement and adherence to treatment. The clinical content was developed in collaboration with both young people and a multidisciplinary team in order to ensure a user-centered approach to the design of evidence-based therapy content to treat SAD symptomatology in young people with FEP.

## Materials and Methods

### Development Aims

The clinical content of the online intervention, termed *EMBRACE*, is based on an integration of the Clark and Wells (20) and Rapee and Heimberg (21) CBT-based treatment models for SAD, which are recommended as best practice in the treatment of SAD by the NICE guidelines (22). We adapted the integrated CBT model to incorporate clinical considerations relevant to a FEP population as identified by the literature, including therapeutic content to address maladaptive shame cognitions, perceptions of lower social rank, and paranoid social–evaluative concerns (10–17).

To maximize interactive usage and adherence to the intervention—in collaboration with a multidisciplinary team of clinicians; young adult fiction writers; a cartoonist; and focus group comprised of young people with the lived experience of SAD, FEP, and serious mental ill-health conditions or any combination thereof—we also developed a number of therapeutic comics. Within the design of the online intervention, social networking elements were also incorporated, including a newsfeed, in which users and moderators will be able to post relevant content, and an online forum, whereby users can interact to help each other problem-solve issues pertinent to social anxiety.

EMBRACE was also designed to be a moderated intervention. Not only do users have the opportunity to interact socially with their peers online, but clinical and peer moderators will also be available to assist young people as they navigate, problem-solve, and make the most of the available evidence-based material.

### Study Design

The EMBRACE intervention is based on the moderated online social therapy (MOST) model pioneered by the eOrygen team at Orygen, the National Centre of Excellence in Youth Mental Health, and developed in collaboration with investigators from The Australian Catholic University and the Department of Computing and Information Systems at The University of Melbourne (45–48). The MOST model is a framework for online interventions in youth mental health that uniquely incorporates i) peer-to-peer online social networking, ii) individually tailored interactive psychosocial interventions, and iii) the involvement of expert mental-health and peer moderators (45–48). The elements of the MOST model have been applied to a world-first online system entitled *HORYZONS* (33, 45), an 18-month randomized controlled trial (RCT) designed to maintain the clinical benefits of early intervention beyond discharge from specialist FEP services. A 6-week pilot study assessing the feasibility, acceptability, and safety of HORYZONS in an FEP population (*N* = 20) showed that HORYZONS is a feasible, safe, and highly acceptable intervention, with findings from the study indicating that 70% of the participants logged on weekly (with a total of 275 logins) and no incidents (i.e., adverse events or inappropriate usage) occurred during the study (45).

The efficacy of HORYZONS as an innovative online intervention for the maintenance of specialized treatment effects in FEP has been recently evaluated *via* a single blind randomized control trial (33). This RCT compared the current model of early intervention for psychosis (i.e., 18 months to 2 years of specialized treatment followed by discharge to standard treatment; FU-ST) and FU-ST in conjunction with HORYZONS, and the results are currently being analyzed (44). The EMBRACE intervention was developed as a purpose-built tailored SAD intervention to be offered to completers of the HORYZONS RCT (at the 18-month follow-up assessment point) who experience social anxiety symptoms as identified by a score exceeding the subclinical threshold on a validated measure of social anxiety (49). These individuals will have the opportunity to take part in a 12-week moderated online social intervention that uniquely incorporates therapeutic comics to target SAD in a FEP population.

### EMBRACE Intervention Design

Firstly, in terms of clinical content, EMBRACE was designed to incorporate 12 independent online modules (which we term *steps*). Each step included clinical content that targeted a particular therapeutic aim related to SAD, and was presented online *via* four formats, including 1) a brief psycho-educational description of each therapeutic concept, 2) unique therapeutic comics designed to maximize engagement with the pathway, 3) discrete behavioral experiments (referred to as *actions*), which were designed to address safety and avoidance behaviors that maintain social anxiety symptomatology, and 4) an interactive discussion forum (referred to as *talking points*) that allowed users to answer a question about related content. The talking point feature was designed specifically to provide users with an opportunity to directly interact and problem-solve topics with their peers (see **Table 1** for a detailed description of these key intervention features).

**Table 1:** Description of intervention features and clinical content of incorporated steps.

Component	Description
**Therapy content**	
**Intervention** **(OASIS)**	Collection of 12 steps conceptually linked together to comprise a 12-week online intervention designed to alleviate social anxiety symptoms.
**Steps**	Steps are composed of interactive therapy modules, each covering a single therapeutic concept and requiring approximately 15 min to complete. In the pathway, all steps were designed to specifically target cognitions and behaviors associated with the maintenance of SAD symptomatology, as per the Clark and Wells model of SAD (20). In addition, three steps were designed to address relevant topics (e.g., intimate relations, self-acceptance, managing negative events) identified *via* feedback from four lived-experience focus groups. Finally, three steps also addressed clinical considerations specifically relevant to a FEP and SAD comorbid population (i.e., addressing shame, social rank cognitions, and paranoia), as identified in the literature (10–17).
**Step content**	
**Key Concepts**	Accessible psycho-educational descriptions of therapeutic concepts and outlines based on the purpose of the particular step for the participants.
**Comics**	Twelve comics (one for each step), each comprising of between 20 to 24 story board panels focusing on a particular therapeutic theme and target related to the treatment of SAD.
**Do Its**	Unique behavioral experiments known as *actions*, where the young person can apply therapy content (e.g., reducing and dropping safety behaviors) to relevant real-world situations, to help foster adaptive coping strategies in participants. The ultimate goal of these actions is to increase practice and the generalization of skills to real-life situations by using context-specific, action-based recommendations (28). Actions were designed as two-tiered hierarchal behavioral experiments, that is, individuals had the option to choose a low-intensity entry-level option (option A) or a high-intensity, more challenging (option B) behavioral experiment. Providing individuals who experience SAD with hierarchal options is an important consideration, given the heterogeneity within anxiety disorders in general (30).
**Talking Points**	Questions are embedded within each of the steps to encourage users to discuss and share their own experiences regarding a specific topic, thereby integrating the social networking component of the intervention and prompting direct interaction.

### The Development of Clinical Content

#### CBT-Based Models of Social Anxiety

All 12 steps of EMBRACE were developed to align with Clark and Wells’s (20) and Rapee and Heimberg’s (21) empirically supported CBT model of social anxiety (22). The CBT model by Clark and Wells (20) places emphasis on addressing maladaptive cognitions and behaviors (avoidance and safety behaviors) associated with the maintenance of social anxiety symptomatology. Clark and Wells (1995) argue that, in addition to information-processing biases about the self (e.g., *I am stupid*), self-focused negative attention derived from internal somatic cues (e.g., sweating, trembling) maintains the cycle of SAD. There is substantial common ground between Clark and Well’s and Rapee and Heimberg’s models of SAD, with more agreements than differences (50). However, Rapee and Heimberg expand on Clark and Wells theory by describing a more interactive relationship between self-monitoring of internal cues and monitoring of the environment for confirmation of external threats (e.g., negative disapproval from others). Integrating these evidence-based treatment models, the theoretical basis of the EMBRACE intervention aims to address the associated maladaptive cognitions and behaviors that socially anxious individuals engage in which perpetuates SAD. In addition to challenging maladaptive cognitive biases associated with SAD, aligned with the Clark and Wells model, the clinical content places emphasis on ways of reconfiguring the affected individual’s processing strategies by maximizing opportunities for disconfirming negative beliefs *via* a direct observation of the social situation (i.e., behavioral experiments), rather than *via* one’s own reflections (leaving one susceptible to cognitive biases). In addition, aligned with Rapee and Heimberg’s model, a further aim of the intervention will be to help socially anxious individuals develop the skills to effectively direct their attention away from an unhelpful mental representation of the self and from scanning their external environment for confirmation of threat.

Empirical findings provide support for both Clark and Wells and Rapee and Heimberg’s CBT-based model of SAD (20, 21). For example, research findings indicate that individuals with SAD perceive ambiguous information in a threatening manner, and numerous studies show that individuals with SAD rate their SA symptomatology (e.g., sweating, blushing) as more prominent than do independent observers rate it (45). Additionally, studies have found that, when recalling anxiety-provoking information from a social situation, individuals with SAD tend to report on events from an external perspective, as if objectively observing or analyzing oneself (51, 52). Finally, there is a great deal of support for the assertion that individuals with SA preferentially direct their attention to threatening information. For example, numerous studies have found that individuals with SAD are slower at color-naming threat-related words in comparison to nonthreat-related words in an emotional Stroop task—a gold standard behavioral measure of selective attention (52, 53).

Research has also shown that when a social-evaluative situation is encountered, negative self-beliefs contribute to the experience of anxiety and an attentional shift to the self and, as a result, other maladaptive beliefs become salient (54). Meta-analytic findings demonstrate support for addressing negative self-beliefs in individuals with SAD as a means to alleviate negative self-focused attention (54). A primary focus of the EMBRACE intervention therefore will be to assist young people gain awareness of negative self-beliefs and to counter irrational thoughts with more realistic compassionate evaluations.

#### Adaption of the Integrated CBT SAD Model for FEP

All 12 steps within the EMBRACE intervention adhere to the integrated CBT model of SAD as previously described. Three of the 12 steps, however, were also specifically adapted to address therapeutic targets relevant to a comorbid SAD and FEP population (as reported *via* current literature findings), including addressing shame cognitions, perceptions of lower social rank, and paranoia (10–17). For example, empirical findings indicate that individuals with social anxiety and a FEP diagnosis experience greater shame attached to their diagnosis and perceive that the diagnosis or mental ill-health categorization places them apart from others (i.e., socially marginalizing them from others and, in turn, incurring a low social status) (11–14, 15, 17). These findings suggest that shame cognitions and social-rank concerns arising from a stigmatizing illness (i.e., psychosis) play a significant role in the maintenance of social anxiety in psychosis (11, 15, 17). Psychological interventions could be enhanced by taking into consideration these idiosyncratic shame and social-rank appraisals when addressing symptoms of social anxiety and associated distress in this particular population. It may be the case that, for some individuals, the shame of mental ill-health and fear of being devalued and rejected by others once the diagnosis is revealed underlies the development and maintenance of social anxiety and avoidance in psychosis (11).

In addition, evidence indicates that paranoid thoughts build upon commonly experienced social evaluative concerns (55–57). Other studies have shown that, in some cases, social anxiety is predictive of the occurrence of paranoid thoughts and the persistence of persecutory delusions (55–59). One objective of EMBRACE, therefore, is to help normalize feelings of paranoia and make it understandable for the affected individuals; it is important, for example, to help individuals who may experience both paranoia and social anxiety to differentiate between the two distinct experiences and help understand their triggers.

#### Comics as an Effective and Engaging Medium

Comics can serve as an effective and unique visual medium to present clinical content relating to social anxiety experiences. Although comic narratives are often thematically similar to standard textual accounts of ill-health, their powerful visual messages convey an immediate visceral understanding in ways that conventional texts cannot (60, 61). There are a number of advantages for utilizing comics in a mental health intervention. Firstly, research findings indicate that not only do young individuals typically prefer information conveyed in images over information presented as text (62–64), but the images can make the content more accessible, engaging, and memorable to youth (64, 65). Graphic stories with their illustrated narratives may also have positive impacts on readers’ engagement, in addition to their memory and conceptual learning (64). For example, research findings (65) have shown that illustrations accompanying text reinforce information in the text, provide coherence, and help establish settings in the narrative, making comics an accessible and engaging means of obtaining complex information.

Comic narratives employ the complex interplay of text and images, which give them the potential to convey concepts effectively and therefore motivate client engagement. It may be the case that for some individuals, visual and narrative illustrations in therapy-based comics would be more helpful than a lengthy explanation by a psychologist or mental health practitioner, as comics have the potential to be understood intuitively, quickly, and comprehensively (66). Comic narratives have not often been utilized in health promotion or online interventions, but they hold great promise as a viable teaching tool, especially among young adults in terms of maximizing engagement potential (66, 67). Emerging research suggests that comic narratives can benefit educational purposes by, for instance, helping struggling younger readers and English learners of any age as they combine pictures and words and give visual cues to explain the text (67, 68). This may be an important consideration within a psychosis population where some individuals may experience cognitive deficits associated with the progression of the illness (69). Comics also have an important fun factor, which young people respond well to, attracting the interest of reluctant participants and eliciting more pleasurable reading in adults who experience cognitive impairment. Comics can also introduce individuals to more abstract concepts (e.g., safety behaviors) or those that are difficult to explain (e.g., flight or fight response).

##### Multidisciplinary Collaborative Process to Design Therapeutic Comics

In collaboration with a multidisciplinary team of mental health workers; fiction writers for youth literature; a cartoonist; and young people with the lived experience of SAD, FEP, and/or a serious mental health condition, we designed 12 unique therapy comics based on the integrated CBT model of SAD to treat social anxiety symptomatology in an FEP population. The multidisciplinary design process began with clinical researchers identifying 12 key themes based on the integrated CBT-based SAD treatment model. The clinical team then sought input from young people with the lived experience of SAD, FEP, and a serious mental ill-health condition, or a combination thereof, *via* two focus groups conducted in 2016, after which the thematic content of the proposed comic was further refined. The proposed therapeutic themes were provided to the writers, who created an initial draft based on the integrated clinical and focus group feedback. The cartoonist then added graphics to the final script, which was reviewed by each member of the collaborative team and finalized by consensus. In 2017, the clinical team again presented samples of the final comics to young people who were divided into two focus groups; their feedback was consolidated, and the comics were further refined.

##### Focus Group Feedback

As discussed, four focus groups (1–2 h in duration) were conducted in 2016–2017, comprising of individuals (*N* = 16) with the lived experience of SAD, psychosis, and serious mental ill-health, or a combination thereof. The focus groups provided feedback on the clinical content of the comics in development, in addition to providing valuable information about topics of interest that should be included in an intervention for young people (e.g., intimacy in addition to peer relationships and SAD). A total of 10 male and six female participants (18 to 25 years of age) comprised these focus groups. Specifically, the focus group participants were asked to provide feedback on 1) proposed therapeutic themes/steps in development, 2) topics that should be addressed relevant to a youth audience, and 3) the therapeutic comics in development, as outlined by the collaborative creative development process highlighted above. The results of the focus groups’ feedback were used to finalize the thematic development of three specific steps, which focused on 1) romantic relationships and social anxiety, 2) self-compassion (and the overlap of perfectionism and social anxiety), and 3) fostering self-acceptance (avoiding personalizing biases and increasing positive emotions). In addition, their feedback was used to further inform the comic design elements (e.g., comic themes, character formation, aesthetics of comics, storylines, etc.).

#### Facilitating Social Networking

The EMBRACE intervention includes social networking elements *via* its newsfeed and talking point functions. The homepage also functions as a newsfeed, demonstrating all relevant activity, and users receive push notification updates relating to peer and moderator posts on the EMBRACE system. The homepage was designed as an online newsfeed so that users, peers, and moderators could post comments, upload information to share (e.g., videos, pictures, stories), and like one another’s content. Therefore, it both enables and encourages direct interaction between online peers. The findings from persuasive technology research show that push notifications and prioritizing newsfeed content that users find valuable (like recent posts from peers) can be an effective means to capture users’ attention and align their usage of the social networking elements with the intervention’s intent (40, 43).

Social interaction is also encouraged within steps through a feature termed *talking points*, which are questions that promote users to discuss and share their own experiences by commenting in the newsfeed. More specifically, talking points allow users the opportunity to freely discuss a set topic relevant to social anxiety experiences *via* a safe, non-stigmatized, online forum feature (e.g., taken from step 7, addressing SAD and perfectionism: *Can you think of a social occasion when your need for things to be perfect was self-sabotaging? What advice would you give a friend in the same situation*)?. Online discussion forums provide an opportunity for social support, and research findings support this, showing their benefits to users’ well-being. In particular, online support can empower young people; help them to develop new online friendships; share personal experiences and communicate with others who understand them; provide information and emotional support; and, most importantly, help them feel less alone by normalizing their experiences in the world (70, 71).

#### Clinical Moderation

The EMBRACE intervention adopts the MOST conceptual model of online interventions and thus provides an interactive psychosocial intervention that is enhanced by a moderated online social networking environment (45). As part of the intervention, clinical moderators will be responsible for moderating the online environment with the purpose of improving clinical functioning. Their role will be to provide guidance, monitor participants’ clinical status, and ensure the safety of the social networking aspects of the online environment. MOST moderation follows a theory-driven model known as *supportive accountability*, which posits that human support enhances engagement through accountability to a moderator who is perceived as trustworthy, benevolent, and having expertise (72). Accountability involves clear, process-focused and user-driven expectations that take into account patient motivation (i.e., the level of support is inversely proportional to the patient’s intrinsic motivation) (72).

In addition to moderating the online environment daily (i.e., 2 h/day during weekdays and 1 h/day during weekends), over a 12-week period, weekly one-to-one client contact between participants and their assigned clinical moderators (*via* phone call or messaging) will be agreed upon as a condition to participation in the intervention. Clinical moderators will be responsible for establishing the initial contact with the participants *via* a phone call to welcome them to EMBRACE, establish a shared formulation, and identify specific goals for treatment. A moderator manual was developed to ensure therapeutic fidelity to the specific weekly goals of the intervention. The manual includes a safety protocol, outlines the specific therapeutic target for each weekly step, and provides examples of weekly communication to be sent to encourage adherence to the intervention, in addition to a problem-solving guide (e.g., to address non-adherence to intervention). In addition to adhering to a moderator manual, moderators will also attend weekly telephone supervision with a clinical psychologist and fellow moderators to discuss clinical issues pertinent to the clinical moderation process of the intervention.

## Results

The content of the 12-step EMBRACE intervention is detailed in **Table 2**, including the therapeutic target for each weekly step and its associated theoretical, empirical, and clinical basis.

**Table 2:** Description of the SA steps, therapeutic target, and its theoretical, empirical, and clinical basis.

Step	Therapeutic target	Theoretical, empirical and clinical basis
1	Identifying situational triggers	CBT model of SAD
2	Identifying and challenging automatic thoughts	Integrated CBT model of SAD
3	Unhelpful self-focused attention	Integrated CBT model of SAD
4	Acceptance of physical sensations	Integrated CBT model of SAD
5	Overt avoidance behaviors	Integrated CBT model of SAD
6	Covert safety behaviors	Integrated CBT model of SAD
7	Fostering intimate relationships (i.e., targeting safety behavior of concealment)	Focus group feedback
8	Self-acceptance (i.e., targeting maladaptive perfectionistic cognitions)	Focus group feedback
9	Managing negative events in everyday life (i.e., targeting personalizing bias)	Focus group feedback
10	Shame (cognitions) and associated safety behaviors	Empirical findings relevant to psychosis and comorbid SAD^1^
11	Paranoia (cognitions) and associated safety behaviors	Empirical findings relevant to psychosis and comorbid SAD^2^
12	Social rank (cognitions) and associated safety behaviors	Empirical findings relevant to psychosis and comorbid SAD^3^

^1^Substantial empirical evidence shows that a psychotic disorder diagnosis carries severe social stigma, and many of those diagnosed internalize this stigma and suffer shame and diminished self-esteem (12–13, 14, 17). ^2^Empirical findings suggest that paranoid thinking and social anxiety can overlap significantly, and issues of social power/rank may underpin both forms of anxiety (13–14, 15, 57). ^3^Study findings show that individuals with SAD tend to assess their worth based upon how they rank in comparison with others (i.e., social rank) (15, 17), resulting in a view of the self that is highly linked to the views of others. For individuals with psychosis and SAD, this relationship is further complicated by feelings of shame, social rejection, and entrapment associated with the stigma of schizophrenia.

### Description of the Finalized Intervention

The moderated online social intervention begins with a welcome message to participants, outlining the purpose of the 12-week intervention and providing a description of its key features. After reading the welcome/introductory message, the participant clicks *next* to access the first of 12 steps. The first component of a step includes what we have termed *key concepts*, which aim to orientate the participant to the purpose of the weekly step and provide a psycho-educational explanation of the potential benefits and barriers of the step (i.e., outlining the benefits of participating in the step and addressing a multitude of possible barriers to participation).

#### Therapeutic Comics

**Figure 1:**
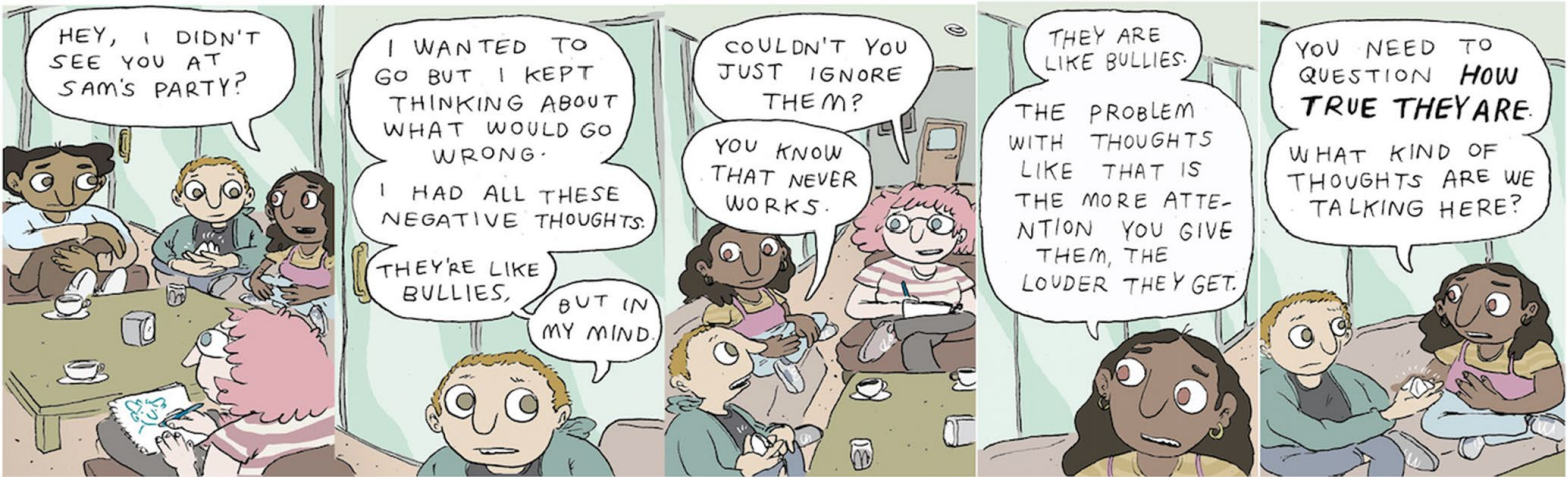
Therapeutic comic panels.

The second key component of a step is the therapeutic comic, which narratively addresses the experience and effective management of a particular social anxiety symptom in an engaging and compelling way. There are a number of additional benefits to using online comics as a SAD treatment modality in FEP, including the following three key points: 1) visual appeal (73, 74): the natural human attraction for images allows comics to capture and hold the interest of the reader; 2) fixed images (73–76): the permanent visual component of comics as opposed to movies and animation, in which the medium dictates the speed at which the vision progresses, allows reading to progress at the reader’s pace. Additionally, the amount of time granted to review, assimilate, and assume the role or behavior shown is unlimited; 3) user-friendly format (73–76): comics can serve as an intermediate step towards difficult concepts, offering a less challenging approach for readers with cognitive difficulties or those reluctant to tackle a particular topic. **Figure 1** provides an example of 5/24 panels of a comic titled *Faulty Fortune-Telling*, developed to address maladaptive cognitions associated with SAD (step 2 of the 12-step intervention).

#### Actions/Behavioral Experiments

The third key component of a step is an action or behavioral experiment, a cognitive strategy in which a maladaptive cognition is tested through a real-life experience. Research findings suggest that behavioral experiments may be effective because they use a real-life experience that shares many functional similarities with exposure, but are explicitly framed as a test of a key cognition, so they blend propositional and experiential learning (77). For example, the findings from a 2010 meta-analysis that examined the efficacy of behavioral experiments, compared to exposure in social anxiety treatment, suggest that behavioral experiments may be more effective than exposure in which this test of cognition does not occur (77).

**Figure 2:**
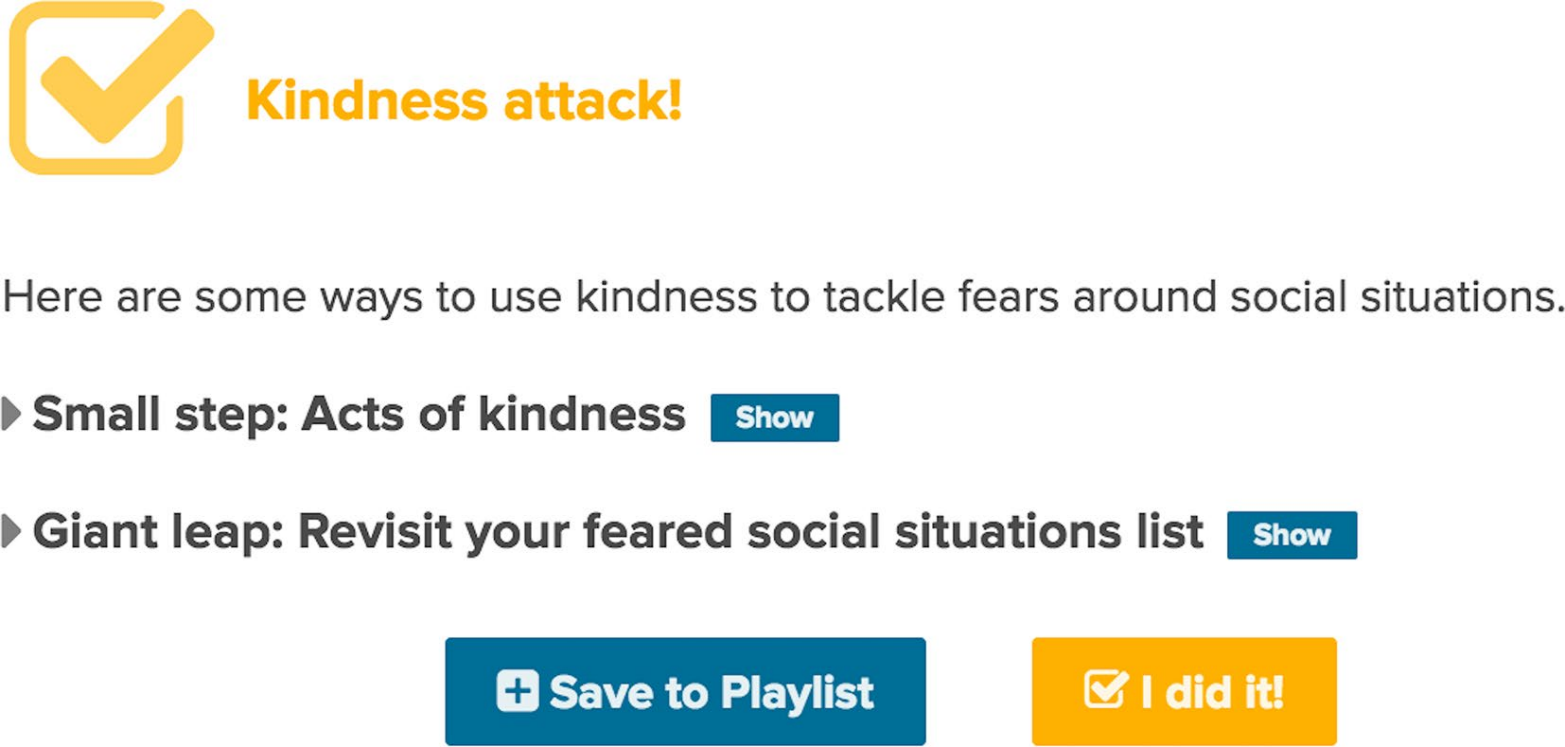
Behavioral experiment options.

The behavioral experiments for each of the 12 steps were designed as two-tiered hierarchal stages involving a low-intensity behavioral experiment titled a *small step*, intended to create awareness, and a higher intensity *giant leap* option, which involved experimentation, namely through planned activities, to test existing beliefs and/or help to test more adaptive beliefs in real-life scenarios. **Figure 2** provides an example of how these options would be presented to a participant *via* the EMBRACE intervention.

#### Talking Point

The fourth and final key component in a weekly step is a talking point, a question that offers the opportunity for young people to use a dedicated online social forum to discuss an issue relevant to experiencing and/or managing social anxiety with their peers. Research findings suggest that online forums that assist discussion among peers provide young people with both informational and emotional support in both directive and nondirective ways (78). The nondirective approach refers to young people providing others with support by sharing their own experiences. These posts do not include explicit advice to act in a particular way; instead, the sharing process is posited to be helpful to the individual posting the content (78, 79). The directive approach, in contrast, involves individuals giving the poster an explicit suggestion. Knowing others have been or are currently going through a similar situation provides a normalizing experience and provides the opportunity to share experiences and feelings with other peers (78).

**Figure 3:**
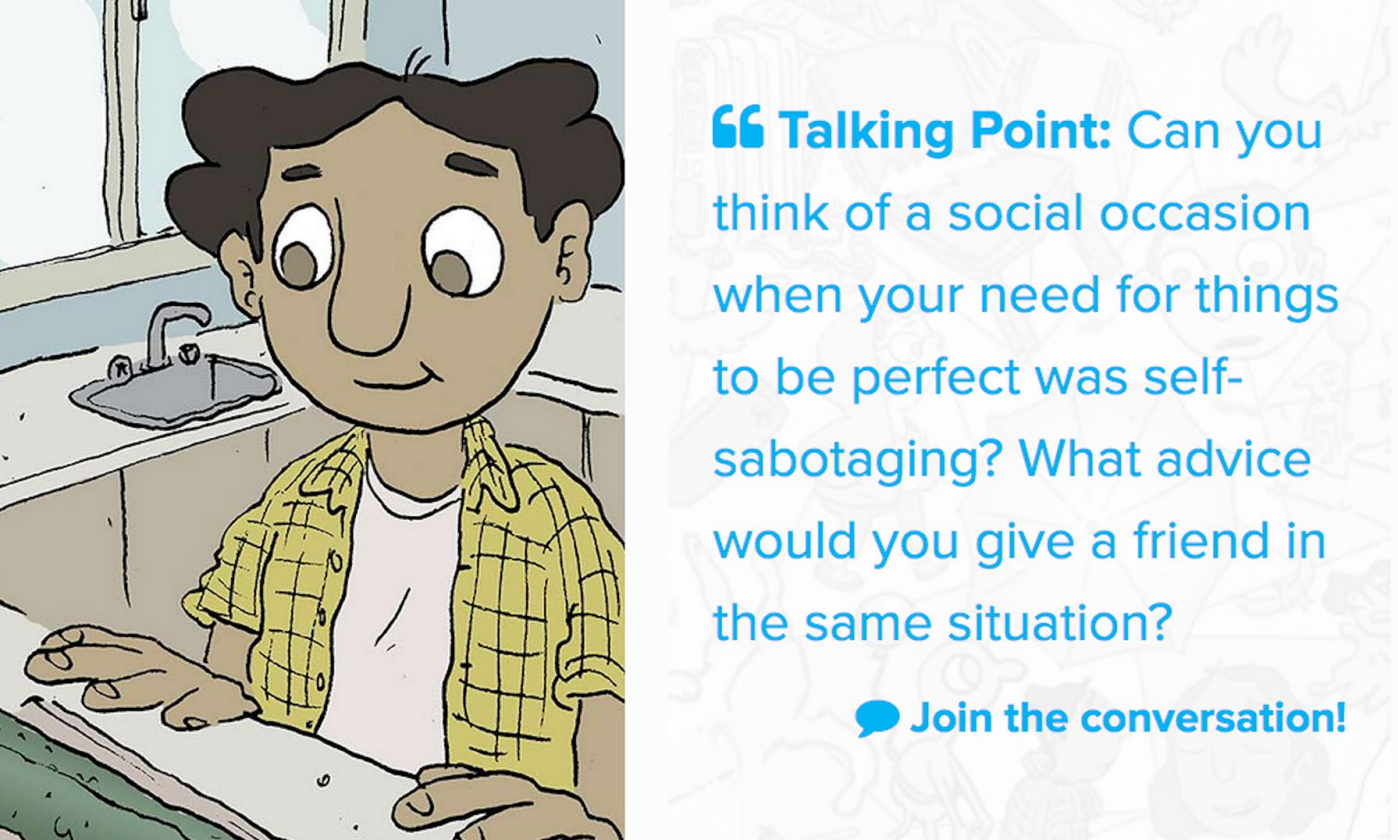
Talking point.

Sharing opinions and experiences on given topics related to the pathway content also has the potential to provide a private and emotionally supportive environment; this is especially so for forums moderated by clinicians (79, 80). Furthermore, online forums may be particularly appealing to young males who tend to have lower levels of help-seeking behavior than their female counterparts (80). **Figure 3** provides an example of how a talking point would be presented *via* the EMBRACE intervention—joining the conversation allows the participant to join the particular online forum related to the given talking point.

## Discussion

### Key Findings

Growing empirical findings indicate that comorbid SAD constitutes a significant issue for individuals diagnosed with a psychotic disorder (6). There is also growing evidence that SAD is a relatively common occurrence following a FEP (10, 17). Indeed, findings suggest that, in the first year following the onset of psychosis, 29% of young people and adults (individuals aged 16–35 years) are likely to experience symptoms that meet the clinical diagnostic criteria for SAD (10, 17). Research findings also show that experiencing SAD in the early stages of psychosis is associated with poor premorbid functioning, depression, and a reduced quality of life (81). Yet to date, there has been limited research or clinical focus on the treatment of co-morbid SAD in psychosis with only two RCTs (23, 24) conducted to date, which have examined the efficacy of CBT-based interventions to treat SAD as a primary concern for individuals with psychosis. To date, there have been no studies conducted that examine the treatment of SAD in a FEP population. This is concerning given that evidence supports early intervention to produce better symptomatic and functional recovery outcomes at a critical stage of ill-health (26).

This paper aims to address this limitation, and presents the design and development of an innovative, engaging, moderated internet-based intervention to treat SAD in young people with FEP. Two empirically supported CBT-based models of SAD were integrated to form the basis of the clinical content for this 12-week intervention. The integrated CBT-based model of SAD was then adapted to incorporate clinical content particularly relevant to a FEP population, including addressing maladaptive shame cognitions, perceptions of lower social rank, and paranoia. The EMBRACE intervention uniquely utilizes engaging therapeutic comics, designed in collaboration with a multidisciplinary team *via* a highly collaborative process. The intervention also utilizes a unique moderation model in which clinical moderators will maintain weekly contact with users, adhere to a moderator’s manual to ensure fidelity of weekly treatment goals, and will attend weekly group supervision with a clinical psychologist to problem-solve issues and discuss methods to tailor content to the participants (e.g., suggesting therapeutic content relevant to a particular user *via* the newsfeed). It is hoped that the intervention when implemented will provide users with the opportunity to test cognitive assumptions in a collaborative safe online environment. In addition, users will be encouraged and coached by clinical moderators to test their cognitive assumptions in an applied setting *via* a variety of tailored discrete behavioral experiments, thus generalizing their treatment gains.

### Potential Shortcomings and Limitations

Internet-based interventions offer specific advantages and disadvantages in the treatment of SAD. For example, being able to limit communication *via* email or messaging, rather than *via* face-to-face, is likely to lower the help seeking threshold (82, 83). At the same time, this may be seen as a shortcoming in the treatment of SAD, as it has the potential to facilitate avoidance of direct face-to-face contact and, in doing so, the experience to overcome socially anxiety-provoking situations (36, 84,). It is anticipated that the weekly communication with clinical moderators will provide EMBRACE users with an opportunity to engage in a process of change that will ultimately reduce avoidance of real-world social interactions. A systematic review examining internet-based interventions for SAD indicated that online CBT interventions are effective in reducing social anxiety symptoms, with treatment gains remaining stable after treatment termination (36, 83, 86). The results of internet-based CBT trials, moderated by clinicians, have shown results comparable to those achieved in face-to-face CBT for SAD (84, 85, 87).

The therapeutic alliance is another factor that is widely regarded as important in psychological outcome research (85). Several studies have examined the therapeutic alliance *via* online interventions, and most studies have demonstrated no association with outcomes, even when the alliance ratings were high (84, 85, 87). However, study findings have also shown that the alliance early in the intervention predicted the outcome (87); therefore, the relationship between the clinical moderators and the participants may inadvertently affect the reported outcomes—a consideration for future research examining the efficacy of the EMBRACE intervention. However, to address this potential limitation, the moderator’s manual was specifically designed to increase treatment fidelity and reduce heterogeneity in clinical moderators’ therapeutic approaches. Likewise, the influence of other hypothesized agents of change in CBT interventions, such as conducting behavioral experiments or modifying self-focused attention, has not yet been analyzed in the internet-based setting (86, 88). Future research should make use of the great potential of internet-based studies to recruit large patient samples and to create records of participant and clinician behaviors.

Another potential shortcoming in regard to the development of the EMBRACE intervention is the recruitment of young people for the focus group’s feedback. The focus groups were comprised of individuals with a psychotic disorder and/or SAD diagnosis, in addition to other individuals with an unspecified clinical mental ill-health diagnosis. As a consequence, the feedback was not limited to the experiences of young people with a lived comorbid SAD and psychosis experience. While this may be seen as a limitation, it can also be interpreted as a strength because one of the main aims of the focus groups was to gather feedback on which topics would be most relevant to include in the mental health intervention in terms of a youth perspective, and we did this by obtaining feedback from a wide representative sample of youths engaged in clinical services.

### Future Directions

EMBRACE, the moderated online social intervention, was designed to treat SAD in individuals with a FEP diagnosis. The development of its clinical content was based on an integrated CBT model of SAD, which incorporated evidence-based material by both Clark and Wells (20) and Rapee and Heimberg (21); both models are recommended by the NICE guidelines as best-practice in SAD treatment. The process of tailoring this evidence-based content to a comorbid SAD and FEP youth population involved incorporating findings from relevant psychosis literature and adapting the content to include the management of maladaptive shame cognitions, perceptions of lower social rank, and paranoia. The development of unique innovative therapeutic comics to supplement clinical content was done in collaboration with a highly multidisciplinary team of clinicians, young adult literature writers, a comic artist, and several focus groups to ensure user-centered content. The EMBRACE intervention also incorporates a unique moderation model, comprised of weekly contact between moderators and users; in addition, moderators will attend weekly clinical supervision to problem-solve clinical issues and/or gain consensus on tailoring clinical content to the specific needs of the user (e.g., suggesting specific content *via* the online newsfeed).

The EMBRACE intervention was hosted on the MOST platform, which is a framework for online interventions in youth mental health that uniquely incorporates i) peer-to-peer online social networking, ii) individually tailored interactive psychosocial interventions, and iii) the involvement of expert mental-health and peer moderators (33, 45). It demonstrates key advantages over face-to-face therapy, including low costs, high reach (low threshold for engagement), and anonymity (46, 89). The MOST platform also affords us the ability to utilize persuasive technology elements, such as dialogue, social, and credibility support as intervention features (44), which help to maximize user engagement potential and adherence to treatment. Further work is required to test the acceptability, feasibility, safety, and preliminary effectiveness of this novel innovative intervention *via* a pilot trial.

## Conclusion

In summary, we believe that we have developed an innovative, unique, engaging, online intervention that incorporates evidence-based CBT therapeutic content, social networking elements, and clinical moderation to help treat social anxiety as a primary treatment concern in young people with a FEP. The initial feasibility, acceptability, and safety of the EMBRACE study is currently being tested *via* an 8-week single group design, and its results will be published elsewhere. The efficacy of the intervention will need to be tested *via* an RCT prior to its potential dissemination. It is anticipated that the ENABLE intervention will provide young people with a safe supportive online community where they can learn adaptive ways to connect with their peers, manage their social anxiety symptoms, and in doing so *embrace* their true authentic selves.

## Data Availability

The raw data supporting the conclusions of this manuscript will be made available by the authors, without undue reservation, to any qualified researcher.

## Author contributions

CMcE, MA-J, MHL, AK, SR and JG significantly contributed to the development of EMBRACE. SH and PR contributed to the development of comics, whereas SD and CM contributed to the technical design of the online intervention. CMcE wrote the first draft of the manuscript. All authors contributed to and have approved the final manuscript.

## Funding

CMcE is funded by an Australian Government Research Training Program Scholarship. MA-J was supported by a Career Development Fellowship (APP1082934) from the National Health and Medical Research Council (NHMRC).

## Conflict of Interest Statement

The authors declare that the research was conducted in the absence of any commercial or financial relationships that could be construed as a potential conflict of interest.
